# Editorial for the Special Issue: Trends in Population Genetics and Identification—Impact on Anthropology

**DOI:** 10.3390/genes15111387

**Published:** 2024-10-29

**Authors:** Verónica Gomes, Luísa Azevedo, Antonio Amorim

**Affiliations:** 1 i3S—Instituto de Investigação e Inovação em Saúde, Universidade do Porto, 4200-135 Porto, Portugal; 2 IPATIMUP—Institute of Pathology and Molecular Immunology, University of Porto, 4200-135 Porto, Portugal; 3 UMIB—Unit for Multidisciplinary Research in Biomedicine, ICBAS—School of Medicine and Biomedical Sciences, University of Porto, 4050-313 Porto, Portugal; 4 ITR—Laboratory for Integrative and Translational Research in Population Health, 4050-600 Porto, Portugal

## Introduction

Technological advances in genetics have revealed many aspects of human ancestry as well as how our genome was shaped by new environments encountered during ancient migrations. However, our quest to uncover more detail of our journey continues. With an open spirit, the editors of this Special Issue embraced the challenge of gathering the most relevant trends in population genetics and human identification. We welcomed contributions that addressed genetic and genomic diversity at both the population and the anthropological level. The submissions received guided us across the evolution and diversity of human populations. 

The Y chromosome and mitochondrial DNA (mtDNA) have proved to be powerful tools in population genetics and forensic science, as well as in evolutionary studies, as they determine male and female lineages, respectively. Regarding the Y chromosome, Y-STRs and Y-SNPs are the most commonly used markers in population studies. Due to the high mutation rate of Y-STRs, these markers are useful for dating and discriminating lineages, and also for providing information about lineage relationships. Focusing on these markers, Adnan and collaborators [1] explored the male genetic pool of the Gypsy population from Pakistan to understand their origin and determine whether the European Roma people originated from Pakistan. In this study, the Y-STR system used (GoldenEye Y20 system Kit) exhibited high discriminatory power in the Pakistani Roma population, and this population displayed low genetic distances from European Roma populations. Moreover, the migration models used indicated a dominant gene flow from Pakistan to India and from Europe to Pakistan.

In another study, using the Yfiler^®^ Plus PCR Kit, which allows the genotyping of 27 Y-STRs, Ashirbekov and colleagues [2] enlarged the national genetic database of Kazakhstan and Uzbekistan. This enhancement will be relevant for forensic and kinship casework. Additionally, they observed low genetic distances between the western Kazakh tribes of Kazakhstan and Karakalpakstan, indicating a common origin of these populations.

Unlike STRs, which mutate rapidly, the low mutation rate of Y-SNPs allows for the comprehensive tracing of paternal lineages over long timescales. Rodrigues and collaborators [3] used a combination of Y-SNP and Y-STR typing to determine the origin of the paternal lineages in the population of Tierra del Fuego (Argentina) in order to evaluate the impact of migration and admixture processes. The authors found that Tierra del Fuego exhibits high haplotype and haplogroup diversity, with a majority Eurasian contribution in its male gene pool. Focusing on Haplogroup R-M269, the most frequent European haplogroup in this Argentinian population, the results suggest a major influx from Iberia, followed by Italy.

mtDNA genotyping is very useful for tracing back maternal haplogroups. In the light of this, Branco et al. [4] applied approximate Bayesian computation to mtDNA data to evaluate the best-fitting evolutionary scenario and to understand the impact of the Last Glacial Period (LGP) on the genetic background of current Southeast Asian populations. Their analyses revealed that the low sea levels caused by the LGP facilitated active human migrations, thereby increasing the mtDNA diversity in Southeast Asian populations. The authors concluded that, in general, environmental factors should always be considered in order to properly understand human evolution.

With a focus not only on the genetic background of the Roma, mainly derived from evidence gathered by the mtDNA and Y-DNA markers, but also from the clinical and physiological implications of their history in their genomes, Ena and collaborators [5] have provided us with an excellent review on the topic that allows us to better understand the relationship between human evolution and demography as well as the current risk with respect to certain Mendelian and multifactorial diseases (type 2 diabetes, cardiovascular disease, obesity, etc.).

The genetic diversity at the LPL gene locus was analyzed in a Kuwaiti population [6]. The authors evaluated the role of intronic markers in relation to LDL levels, body mass index, and the risk of coronary heart disease, finding associations for these three phenotypic characteristics. For example, the minor alleles of rs274 and rs294 exhibited a protective effect against coronary heart disease. Overall, this study opens up a new dimension on the impact of apparently neutral markers on disease risk. The next step will be to evaluate these results in other human populations.

The estimation of mutation rates for different genetic markers has been challenging, as available data often underestimate the true values. To understand the dynamics of the mutation rate calculation, the study of Antão-Sousa and collaborators [7] has shown that the underestimation of autosomal STR mutation rates is dependent on the allelic frequency spectrum. To enhance the utility and widen the use of their approach within the scientific community, the authors provided a tool (Incomp2Mut) to enable the accurate estimation of autosomal STR mutation rates.

Some genomic markers exhibited a high mutation rate and are known as rapidly mutating (RM) markers. Due to their intrinsic characteristics, their discriminatory power is particularly high, which is of the utmost importance when analyzing consanguineous male lineages. Nazir and coauthors [8] analyzed a sample from Pakistan using a panel of 13 RM Y-STR markers and demonstrated their effectiveness, showing an increased differentiation rate of male lineages as compared with conventional STRs.

This Special Issue also includes a systematic review [9] that discusses the available literature on the hominin history in Wallacea and Sahul populations. This work aims to determine the timing and origin of admixture events with extant hominins, such as the Neanderthals and Denisovans. The authors’ conclusion highlights that, despite significant insights obtained over the last few years, unraveling the complex ancestry of these populations will require additional data from ancient DNA investigations. The coming years are expected to bring fascinating new discoveries on this topic.
